# Pea Pod Valorization: A Green Processing Route to Obtain Cellulosic Reinforcements for Compression Molded Polylactic Acid Biocomposites

**DOI:** 10.3390/ma18194608

**Published:** 2025-10-04

**Authors:** Daniela Negrete-Bolagay, Victor H. Guerrero, Salomé Galeas, Jennifer Tejedor, Patricia I. Pontón, Anja Dosen

**Affiliations:** 1 Department of Materials, Escuela Politécnica Nacional, Quito 170525, Ecuador; daniela.negrete@epn.edu.ec (D.N.-B.); salome.galeas@epn.edu.ec (S.G.); jennifer.tejedor@epn.edu.ec (J.T.); patricia.ponton@epn.edu.ec (P.I.P.); 2 International Centre for Diffraction Data, 12 Campus Blvd, Newtown Square, PA 19073, USA; dosen@icdd.com

**Keywords:** cellulose reinforced, extrusion, compression molding, mechanical properties

## Abstract

The valorization of agroindustrial residues represents a sustainable alternative in the production of materials attractive for sustainable technologies. In this work, cellulosic materials were isolated from treated pea pods aiming to obtain highly crystalline, thermally stable reinforcements for biocomposites. Four different treatments were evaluated; two employed 0.5 or 0.75 M oxalic acid (OA) solutions at 90 °C, and two used 5% *w*/*v* KOH solutions after each OA treatment. The cellulosic materials (10, 20 wt.%) were compounded with a polylactic acid (PLA) matrix and polyvinyl alcohol (0, 2.5 wt.%) as a compatibilizer by extrusion. Compression molding was used to obtain samples to study the composite’s mechanical and thermal behavior. The cellulosic materials and the composites were characterized by Fourier transform infrared spectroscopy, thermogravimetry, and calorimetry. The composites were also subjected to flexural, thermo-mechanical, and water absorption testing. The cellulosic reinforcements obtained using 0.75 M OA and 0.5 M OA and KOH showed the highest crystallinities (91–92%). In general, 20 wt.% reinforced composites showed lower thermal expansion and higher water absorption than those incorporating 10 wt.% reinforcements. The composites incorporating 10 wt.% of 0.5 M OA treated pea pods exhibited flexural modulus/strength 17/3% higher than that of PLA. The composites incorporating 20 wt.% of 0.5 M OA and KOH-treated pea pods showed the highest flexural modulus/strength, 35/25% higher than that of PLA. These results show that agroresidues treated with low-concentration organic acids can be effectively used to tune the mechanical, thermal, and water absorption behavior of biodegradable composites.

## 1. Introduction

Despite their versatility, durability, low cost, and wide array of convenient combinations of mechanical, thermal, and biochemical properties, petroleum-based plastics have tremendously deleterious impacts on the environment. These impacts are related to the pollution associated with their continuously increasing production, extremely slow degradation, deficient waste management, a small portion recycled, little awareness about these problems, and insufficiently effective public policies to mitigate them [1]. Developing a circular plastic economy constitutes an ideal approach to deal with the potentially negative effects stemming from the use of conventional non-biodegradable plastics. In this economy, renewable resources (e.g., biomass, oils) are used to synthesize biopolymers for product manufacturing. At the end of the intended life of these products, they continue through a planned circular end-of-life scenario, including advanced recycling, composting, and upcycling [2]. Developing thermoplastic biocomposites made using biodegradable bio-based polymers and reinforcements represents a currently viable alternative within this framework. Moreover, these biocomposites can conveniently replace traditional plastics in some applications, particularly if low-cost, widely available, easy-to-process, renewable raw materials and residues are used [3].

Polylactic acid (PLA) is a commercially available biodegradable hydrophobic bio-based polymer with good mechanical strength and stiffness, thermal plasticity, and biocompatibility. Despite its high potential, PLA exhibits some disadvantages, including brittleness, low melt strength, heat distortion temperature, and impact resistance, which limit its suitability for various applications [4]. On the other hand, several particulate and fibrous materials, derived from natural resources, have proved very effective for improving the performance of biopolymers such as PLA, especially considering that many of them have low costs (i.e., kenaf fiber: 0.38 kg/USD) and exhibit high specific strength and stiffness [5,6]. Particularly, cellulose is the most abundant natural biopolymer, which can be obtained from lignocellulosic biomasses, including woody forest resources, and widely available agroindustrial residues [7]. Due to its biocompatibility, low density, high specific modulus, biodegradability, and non-toxicity, cellulose has been considered a good reinforcement for brittle thermoplastic matrices such as PLA, improving the mechanical properties of the resulting biocomposites [8,9,10]. Additionally, productivity and cost competitiveness can be increased if these materials are processed using high-throughput, well-established manufacturing techniques such as extrusion, compression, injection, and blow molding. Likewise, if these biocomposites can be adequately managed at the end of their useful life and are biodegradable, recyclable, or compostable, their environmental impact could be minimized while increasing their lifecycle efficiency [11].

Several cellulosic materials have been used as biocomposite reinforcements, including wood powder, natural fibers, lignocellulosic particles [12], and nanosized cellulose [13]. Recent advances in PLA-based biocomposites have focused on the incorporation of plant-derived cellulosic reinforcements, often sourced from agricultural residues such as sugarcane bagasse, pineapple leaves, water hyacinth, and cereal straws [14,15,16,17]. Reinforcements vary in size and morphology, ranging from microparticles to nanofibers and nanocrystals, with loadings typically below 20 wt.% [18,19]. High cellulose content is generally preferred for improving reinforcement yields and enhancing the mechanical performance of the biocomposites. Nanocellulose becomes a good option for various applications since it is possible to improve the biocomposite processability and mechanical performance using smaller reinforcement amounts. However, nanocellulose production requires energy-intensive chemical and mechanical treatments with significant environmental impacts [20]. Moreover, effective dispersion in the matrix is critical, with strategies including the use of polyethylene glycol carriers or surface functionalization of nanocellulose [21]. Although some recent studies suggest that retaining certain amounts of hemicellulose or lignin in lignocellulosic reinforcements can improve interfacial behavior and water resistance [22], other approaches focus on isolating high-purity cellulose to enhance crystallinity and thermal stability, as pursued in this work. These challenges include the selection of low-cost biomass sources such as agro-industrial residues that can be efficiently treated and the development of high-yield, environmentally friendly processing routes to obtain cellulosic reinforcements [23]. Given the hydrophilicity and limited thermal stability that characterize cellulosic materials, it is also essential to formulate manufacturing methods that ensure good reinforcement dispersion and low agglomeration, as well as an adequate rheology and limited degradation of the polymeric biocomposite melt [3].

This work aims to use an efficient and economical technique to obtain cellulosic materials that can be effective reinforcements in PLA biocomposites. One of the objectives was to avoid using highly concentrated toxic chemicals, specialized and energy-demanding equipment, and the need for more complicated compounding methods. Pea pods were selected for cellulose extraction due to their relatively high cellulose content, which may reach between 27.3 and 69 wt.%. These values depend on several factors, such as extraction techniques, sample preparation, plant variety, and growing conditions [24,25,26]. In addition, pea pods show a widespread availability worldwide, with an annual production estimated at 4 million tons, which are often not efficiently utilized [27]. Oxalic acid (OA) was chosen among the organic acids used to obtain cellulose reinforcements since it allows relatively high yields and crystallinities [23]. OA was also combined with an alkaline treatment to isolate cellulose. Even though this extraction process is not completely environmentally friendly, minimizing the concentrations of the acid and the base used reduces the impacts compared to traditional methods. The results obtained show promising valorization alternatives for agroindustrial residues such as pea shells, contributing to global sustainability and fulfilling the principles of the circular economy.

## 2. Materials and Methods

### 2.1. Materials

Pea pods (Pisum sativum) were collected from a market located in Toacaso (0°45′0″ S and 78°40′60″ W), province of Cotopaxi, Ecuador. The shells were washed with tap and distilled water to remove impurities. Then, the agroindustrial residue was dried in an oven at 80 °C for 24 h. Finally, the pea pods were ground using a knife mill Thomas Wiley model 3379-K05 (Swedesboro, NJ, USA) and sieved (100 mesh).

### 2.2. Cellulose Extraction

Four different treatments were used to extract cellulose from ground pea pods. The first two consisted of treating the residues with 1400 mL of oxalic acid (OA) solutions at 0.50 M or 0.75 M, corresponding to ratios of 1.8:1 g OA/g pea pods and 2.7:1 g OA/g pea pods, respectively. The mixtures were heated at 80 °C with constant magnetic stirring for 4 h. These treatments were designated CPP1 and CPP2. The other two treatments, designated CPP3 and CPP4, began with the same OA pretreatment described above and were subsequently treated with 5% (*w*/*v*) KOH solutions as a second step to solubilize the pectin and hemicelluloses remaining in the samples [28,29]. In this step, a ratio of 1.67:1 (g KOH per g of treated biomass) was used. The mixtures were stirred magnetically for 7 h at room temperature. Finally, the resulting materials were washed with distilled water until a neutral pH was reached.

### 2.3. Preparation of PLA Biocomposites

First, the PLA was dried at 80 °C for 24 h to remove moisture. The biocomposite components were then manually mixed. Two weight percentages of cellulosic reinforcements were used (10 and 20% *w*/*w*), and a compatibilizer, polyvinyl alcohol (PVA), was incorporated at 2.5% *w*/*w*. The amount of PLA matrix (90, 80, 87.5, and 77.5 wt.%) used in the preparation of the compounds was adjusted according to the component percentages, as detailed in Table 1. The homogenized mixtures were compounded using a corotating twin-screw microextruder (AX Plastics, Diadema, Brazil). The extruder has six heating zones, with temperatures set as follows: T_1_ = 110 °C (feed zone), T_2_ = 150 °C and T_3_ = 150 °C (compression zone), T_4_ = 147 °C and T_5_ = 147 °C (metering zone), and T_6_ = 140 °C in the die. The temperatures fluctuated within ±5 °C. During extrusion, the materials were processed at a screw speed of 140 rpm, with a torque of 15 Nm/cm^2^ and a residence time of 2–4 min. The filaments obtained were cut using a pelletizer. This procedure was followed to obtain biocomposite pellets with all the formulations prepared. Table 1 shows these formulations, divided into four groups, according to the type of treatment used to obtain the cellulosic reinforcement.

The biocomposite pellets were used to prepare testing samples by compression molding. The temperature set in the hot press was 160 °C, the pressure was 1000 psi, and the process lasted 5 min. After the molding process, the fabricated samples were annealed at 120 °C for 4 h. Subsequently, the samples were physically, thermally, and mechanically characterized to evaluate their performance.

### 2.4. Characterization of Cellulosic Reinforcements and Manufactured Biocomposites

#### 2.4.1. Cellulosic Reinforcements

The composition, structure, and thermal behavior of both raw and treated cellulosic materials were studied. The proximate analysis of the raw pea pod lignocellulosic residues was carried out to determine moisture, ash, and volatile matter contents according to ASTM E872-82(2019) [30]. Fixed carbon was calculated by difference. The lignin (ASTM D1106-21) [31], extractives (ASTM D1107-21) [32], and hemicellulose (ASTM D1109-21) [33] contents in the pea pods were determined according to the given standard methods. Cellulose content was calculated by difference. All the tests were performed in duplicate. Regarding the structure, X-ray diffractograms were obtained using an Aeris Research X-ray Panalytical diffractometer (Malvern Panalytical, Almelo, The Netherlands), using copper K-α radiation (λ = 1.54056 Å) under a 40-kV voltage and 15 mA. The diffraction patterns were analyzed using JADE 9.5 software, applying a structureless whole-pattern fitting approach with Pawley refinement [34]. The background was modeled with a polynomial fit, while peak shape parameters,30 scale, offset, and unit cell parameters were refined. Peak angles were constrained and not refined. Additionally, JADE allows incorporating amorphous profiles in the WPF refinement using a five-parameter pseudo-Voigt function, and it can estimate amorphous content from the fitted profile when the nominal density value (ρ) is provided. Fourier transform infrared spectroscopy (FTIR) was performed using a Jasco spectrometer (Hachioji, Tokyo, Japan), model FT/IR-6800 type A, scanning from 650 to 4000 cm^−1^ at 2 mm·s^−1^.

Regarding thermal behavior, thermogravimetric analysis (TGA) was carried out using a TA Instruments TGA Q500 analyzer (New Castle, DE, USA). The samples were heated from 25 to 1000 °C at 10 °C·min^−1^ under a nitrogen flow of 40 mL·min^−1^. Differential scanning calorimetry (DSC) thermograms were acquired using a TA Instruments DSC Q2000 calorimeter (New Castle, DE, USA) with a heating rate of 10 °C·min^−1^ from 25 to 400 °C and a nitrogen flow of 50 mL·min^−1^.

#### 2.4.2. Characterization of PLA Biocomposites

The effect of adding the different cellulosic reinforcements obtained into the PLA matrix was evaluated in terms of structural, thermal, mechanical, and water absorption properties. FTIR spectroscopy of the biocomposites was performed using a Jasco FT/IR-6800 type A spectrometer (Hachioji, Tokyo, Japan) with the accessory ATR-PRO-ONE, scanning from 400 to 4000 cm^−1^ at a speed of 2 mm·s^−1^.

The thermal stability of the composites was studied by TGA. Sample weights ranged from 10 to 30 mg. Samples were scanned from 25 to 600 °C at 10 °C·min^−1^, under 40 mL·min^−1^ nitrogen flow. DSC analyses were performed to determine the glass transition (Tg) and melting temperature (Tm) of the biocomposites. All the samples were scanned from 25 to 200 °C at 10 °C·min^−1^, under a nitrogen flow of 40 mL·min^−1^. A single $c_{c}$ of the composites was determined as follows:

$$c_{c}=\frac{∆H_{m}}{∆H_{m}^{0}{(1-m}_{f})}100\%$$

where $∆H_{m}^{0}$ is the theoretical value of the melting enthalpy (93 J·g^−1^) for 100% crystalline PLA, $∆H_{m}\;$is the melting enthalpy for the sample studied, and $m_{f}$ is the mass fraction of the filler [35,36]. The thermomechanical behavior of the biocomposites was studied using a TA Instruments Q400 TMA analyzer (New Castle, DE, USA). The measurements were carried out in a temperature range from 25 to 120 °C, with a heating rate of 3 °C·min^−1^, and a loading force of 0.05 N.

For the mechanical characterization, flexural tests were performed according to ASTM D790-17 [37]. Five specimens (3.2 × 12.7 × 61 mm) of each biocomposite formulation were tested using a Shimadzu AGS-X universal testing machine (Shimadzu Corporation, Kyoto, Japan), with a test speed of 1.37 mm·min^−1^ and a 51.2 mm distance between supports. The average and standard deviation of the flexural modulus, strength, and strain at failure were calculated for each formulation.

Water absorption tests were carried out according to ASTM D792-20 [38], immersing the specimens in distilled water at 50 °C from 1 to 21 days. At regular time intervals, each specimen was removed from the water immersion bath and wiped with tissue paper to remove surface water and subsequently weighed using an analytic balance. The water absorption was calculated as the percentage increase in the initial weight [39]:

$$Mt\left(\%\right)=\frac{\left(Wt-Wo\right)}{Wo}100\%$$

where *W_t_* is the weight of the sample after a given immersion time and *W_o_* is the initial weight of the sample.

## 3. Results and Discussion

### 3.1. Characterization of the Cellulosic Reinforcements

#### 3.1.1. Composition and Yield

The cellulose, hemicellulose, lignin, and extractive contents in the raw pea pod shells were 37.81, 34.42, 6.25, and 21.52%, respectively. The proximate analysis showed 84.37, 1.34, 8.77, and 5.52% of volatile matter, fixed carbon, ash, and moisture in the pea pods, respectively. The most convenient process and reagents for cellulose isolation are determined by the availability of lignocellulosic residues, their composition, production scale, and product quality. In this work, the treatment of pea husks for obtaining cellulose was based on using OA and KOH. OA is a dicarboxylic acid in which one or both carboxyl groups can react with the hydroxyl groups of cellulose [40]. OA is abundant, biodegradable, and non-volatile and is considered a sustainable acid. The yield of OA depends on the concentrations and the processing routes (chemical or mechanical), which promote the depolymerization of cellulose, affecting its amorphous regions and leading to high yields of crystalline structures [41]. It is worth noting that OA represents a promising alternative to mineral acids such as H_2_SO_4_, HCl, H_3_PO_4_, and HBr, which often cause significant problems, including equipment corrosion, high water consumption, recovery difficulties, and the generation of toxic byproducts [42]. Furthermore, oxalic acid has been shown to exhibit higher selectivity than sulfuric acid, as it promotes more effective hydrolysis of the β-(1,4) bonds present in cellulose [43].

In a study conducted by Xu et al. (2017) [44], the yields and thermal stability achieved in cellulose production were analyzed. The findings indicated that, with oxalic acid, an 85% yield and high thermal stability up to 350 °C were obtained, whereas with sulfuric acid, the yield was 35% and degradation began at 200 °C [44]. Likewise, the use of alkaline treatments (NaOH, LiOH, KOH, Ca(OH)_2_) has been reported to break the bonds linking lignin and polysaccharides [45]. However, alkaline solutions, especially NaOH, induce cellulose aggregation and, due to their amphiphilic nature, fail to dissolve it. From an environmental perspective, this method is unfavorable, as it requires large amounts of water to neutralize the mixture and remove impurities before subsequent transformations [46].

Therefore, the use of less harmful solvents that reduce wastewater generation is necessary. In this regard, oxalic acid has been employed in the controlled hydrolysis of hemicellulose, preventing cellulose degradation when used at low concentrations [42,43]. Moreover, owing to its low solubility at room temperature, oxalic acid can be easily recovered and reused through a simple crystallization process, thereby improving process sustainability and minimizing environmental impact [47].

In this work, the extraction yields of the cellulosic materials obtained from the CPP1, CPP2, CPP3, and CPP4 treatments were 41.14, 33.63, 22.76, and 22.49% (*w*/*w*), respectively, expressed as a percentage of the initial dry mass of the pea pod shells. These values are similar to the ones reported by previous authors who have employed OA and/or KOH to obtain cellulose. For instance, Romrue et al. (2022) [48] investigated the alkaline extraction of cellulose from rice straw (RS), corn cob (CC), Phulae pineapple leaves (PL), and peels (PP). The procedure was based on the use of 1.4% acidified sodium chlorite (NaClO2) and 5% KOH in all samples. The extraction yields of the cellulose obtained were 32.26, 38.18, 16.60, and 9.05 wt.% for RS, CC, PL, and PP, respectively [48]. It is worth noting that the OA concentration used in this work is relatively low compared to the ones used by previous researchers. For example, cellulose microfibers/nanofibers (CMNF) were obtained from bleached eucalyptus pulp by using 25 wt.% (2.94 M) and 50 wt.% (5.88 M) OA aqueous solutions [49]. These relatively low acid concentrations improve the environmental friendliness of the treatments while still allowing for good yields. On the other hand, the use of 5% *w*/*v* KOH reduces the yield but increases the cellulose content by facilitating the removal of hemicellulose, pectin, and lignin still present after the acid treatment step, as verified by FTIR spectroscopy (see Section 3.1.2). The alkaline treatment breaks down natural fibers into individual fibrils, increasing the accessibility of surface hydroxyl (–OH) groups. These –OH groups act as reactive sites that can form hydrogen bonds with the PLA matrix, improving interfacial adhesion, surface tension, and the wettability of the treated fibers [50,51,52]. Although excessively aggressive alkaline treatments can partially degrade cellulose chains or reduce crystallinity, our study using a low concentration did not affect the cellulose structure. The materials obtained illustrate the possibility of using relatively low concentrations of OA to obtain effective reinforcements of a biodegradable matrix.

#### 3.1.2. FTIR Analyses

**Figure 1:**
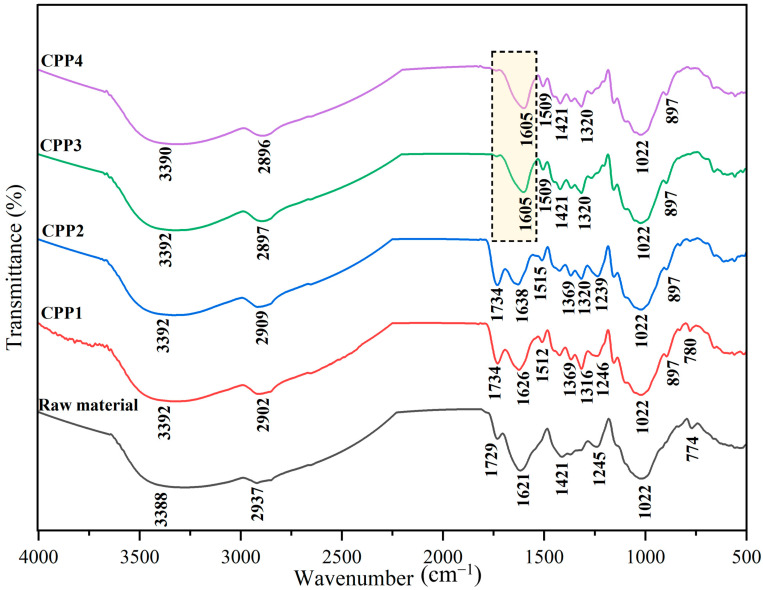
FTIR spectra of cellulosic materials obtained from pea pods by different acid and acid/alkaline treatments.

Figure 1 shows the FTIR spectra for the cellulosic materials obtained through the different chemical treatments studied in this work. The region between 3300 and 3200 cm^−1^ was associated with the –OH groups of cellulose [53]. A broad band related to the vibrations of hydroxyl groups was detected around 3390 cm^−1^. The bands observed around 2900 cm^−1^ represent the characteristic bands of C-H and CH_2_ stretching vibrations in cellulose and hemicellulose.

CPP1 and CPP2 cellulosic materials, observed around 1730 cm^−1^ and 1626 cm^−1^, were attributed to acetyl-uronic ester groups, ferulic, and p-coumaric acids of lignin and hemicelluloses, respectively [54]. These bands disappeared in the CPP3 and CPP4 spectra, indicating the removal of hemicelluloses and lignin through successive chemical treatments [55,56]. In the FTIR spectrum of the extracted cellulose, the region below 1600 cm^−1^ corresponds to the so-called fingerprint region, which contains characteristic vibrations of cellulose [57]. The band at ~1605 cm^−1^ is attributed to –OH bending of absorbed water. Signals at 1421 and 1369 cm^−1^ correspond to the bending vibrations of C–H and C–O groups associated with residual hemicellulose and lignin, respectively. The band at 1425–1442 cm^−1^, assigned to CH_2_ scissoring, is also recognized as the “crystallinity band” of cellulose I, in agreement with XRD results [58]. The absorptions at 1174 and 1120 cm^−1^ are attributed to asymmetric C–O–C bridge stretching, while the band near 905 cm^−1^ corresponds to β-glycosidic linkages between glucose units [59]. In addition, the peaks at 1022 cm^−1^ (C–O stretching) and 897 cm^−1^ (C–H rocking) further confirm the preservation of the cellulose structure after the chemical treatments, as observed in the CPP3 and CPP4 samples [56].

#### 3.1.3. Crystallinity

**Figure 2:**
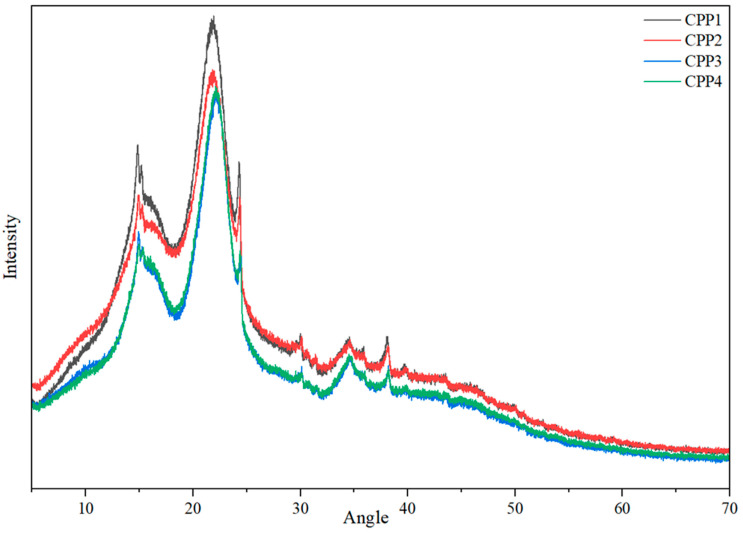
X-ray diffraction (XRD) patterns of the cellulosic materials extracted from pea pods.

The XRD patterns of cellulosic materials obtained from pea pods are shown in Figure 2. The typical pattern of cellulose type I exhibits peaks at 2θ around 15.2, 16.6, and 22.9°, and another main peak at 34.9° [54,60,61]. The cellulosic materials obtained in this work exhibit the same peaks, while the more prominent ones are around 2θ = 14.9° and 22.08°. The similarity of these patterns to those reported for commercial microcrystalline cellulose (MCC), exhibiting peaks at 2θ = 15.4, 16.2, 22.5, and 34.5° [62], indicates that the treatments successfully produced cellulose-rich materials. These diffraction patterns suggest the presence of crystalline cellulose domains, supporting the potential of the extracted materials as reinforcements in composite applications [63].

Although variations in peak intensities were observed between treatments, the broad nature of the diffraction peaks makes it difficult to visually distinguish crystallinity differences between samples. XRD results provide qualitative confirmation of the presence of crystalline cellulose domains in the extracted materials, supporting their potential use as reinforcement in polymer composites.

Broad cellulose peaks indicate small crystallite size and the presence of an amorphous phase. WPF analysis, performed using the PDF-5+ database [64] entry 00-056-1719, showed that CPP1 and CPP2 contain about 35% amorphous content, whereas CPP3 and CPP4 exhibit significantly higher crystallinity. These results suggest that differences in sample preparation and KOH treatment influence the formation of the amorphous phase. Note that a high crystallinity is desired for improving mechanical properties in composite applications [65], suggesting the potential of CPP2, CPP3, and CPP4 as reinforcements.

#### 3.1.4. Thermogravimetric Analysis

**Figure 3:**
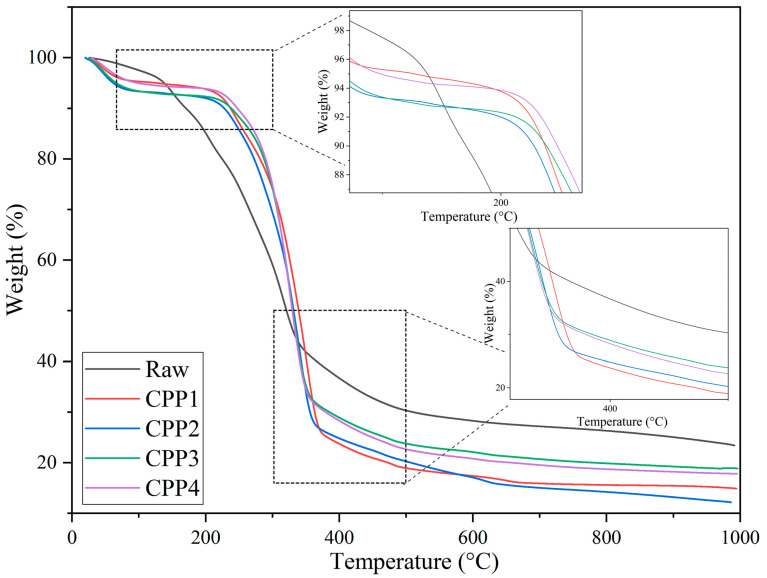
TGA of cellulosic materials obtained after acid and acid/alkaline treatments of pea pod shells.

The thermograms obtained for the cellulosic materials studied are shown in Figure 3, while the onset and maximum degradation temperatures are given in Table 2. The thermograms show a mass decrease around 100 °C due to the removal of moisture and some volatile components from the samples [56,61]. Note that the presence of water absorbed in the samples was also determined in the FTIR spectra shown (Figure 1), where a band around 1630 cm^−1^ was observed. This is due to the hydrophilic nature of cellulosic materials. Around 200 and 430 °C, the degradation of cellulose, lignin, and hemicellulose chains was observed. Note that the glucose units begin to decompose into carbon at lower temperatures, while at higher temperatures, these units depolymerize into volatile products [66]. Above 500 °C, products such as CO_2_, CO, hydrocarbons, and hydrogen leave the samples [67]. The untreated pea pods exhibited lower degradation temperatures compared to the treated ones. This is due to the presence of lignin and hemicellulose in the raw pea pods. The results obtained after treatment demonstrate that cellulose is thermally stable up to 280 °C, where no significant mass loss was detected. Similar results were observed in the extraction of MCC derived from pea pods, where the T_onset_ was 265 °C, and T_max_ was 340 °C [56].

#### 3.1.5. DSC Analysis

**Figure 4:**
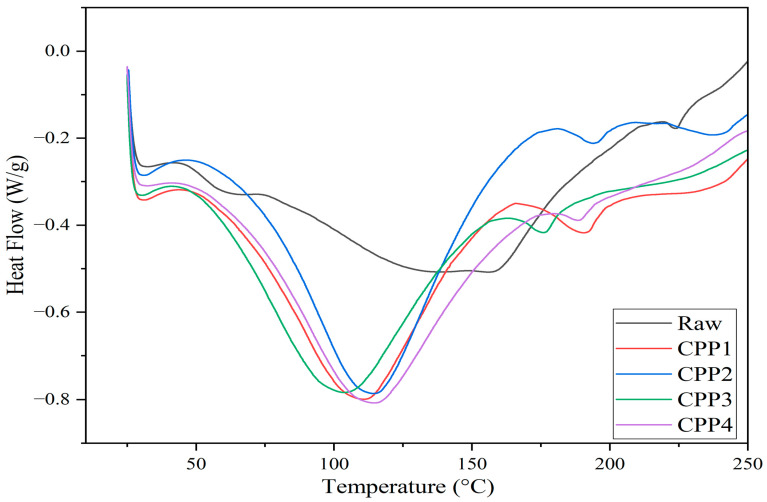
DSC of raw pea pods and treated cellulosic materials.

Figure 4 shows details of water evaporation from the cellulose surface and the initial thermal decomposition of the cellulosic materials. DSC analysis revealed a broad endothermic peak around 50–150 °C for all samples, corresponding to the vaporization of the moisture absorbed due to the hydrophilic nature of the hydroxyl surfaces on cellulose [68,69]. Changes in the enthalpy associated were also observed. The enthalpy change is lower for raw pea pods and increases after the treatments. In the case of the endothermic peak, it shifts to a lower temperature compared to the raw pea pods. This could be explained by an increase in the length/diameter ratio of the cellulosic fibers during the KOH treatment, which facilitates the evaporation of moisture [70]. Similar results were observed when cellulose fibers were modified using vinyltrimethoxysilane and maleic anhydride to improve their wettability in the hydrophobic polymeric matrix. In that case, the changes observed by DSC were also attributed to the length/diameter ratio of the fiber [71].

Summarizing, FTIR spectroscopy and XRD show that the cellulose obtained through the treatments performed has a type I structure. The acid-alkaline treatments effectively removed hemicelluloses and lignin from the pea pods while preserving the cellulose structure, as shown by FTIR spectroscopy. This also increases the crystallinity of the treated materials, as evidenced by XRD.

### 3.2. Biocomposites Characterization

#### 3.2.1. FTIR Analysis

**Figure 5:**
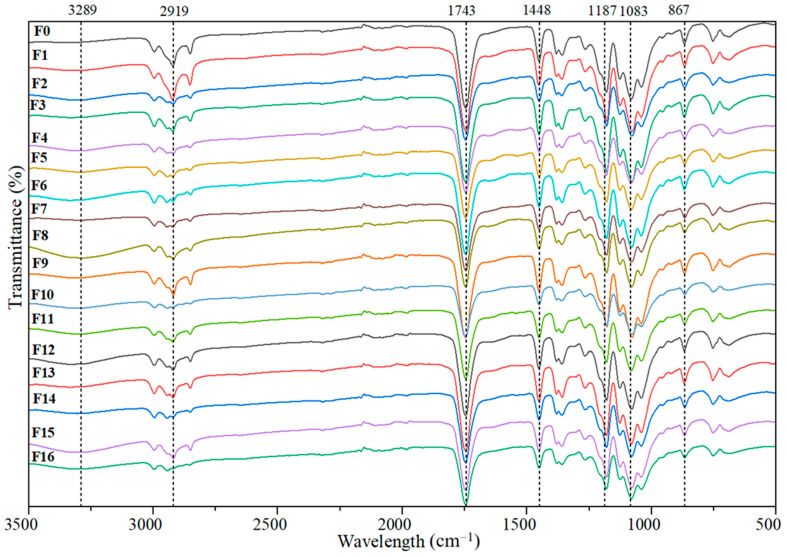
FTIR spectra of the PLA and the biocomposites prepared.

Figure 5 shows the spectra of the PLA matrix used and the biocomposites formulated in this work. The characteristic absorption bands of pure PLA were observed at 1448, 1187, 1083, and 867 cm^−1^. The stretching vibration of the C-O groups corresponds to the bands at 1187 and 1083 cm^−1^. The bands observed at 1448 cm^−1^ could be related to the deformation of carbon-hydrogen (C-H) bonds in the CH_3_ group. Likewise, bands at 920 and 867 cm^−1^ appear due to the presence of C-C single bonds [10].

Regardless of the cellulosic reinforcement content, the position of the bands does not change. On the other hand, the bands around 3300 and 2850 cm^−1^ are attributed to the stretching of -OH and -CH groups, characteristic of cellulose. This indicates that the cellulosic reinforcements and the PLA matrix are interacting. In the band at 1743 cm^−1^, no change was observed after the incorporation of cellulosic reinforcements. This is due to the interaction between the carbonyl group (C-O) of PLA and the hydroxyl groups (-OH) of cellulose [72]. Furthermore, the presence of coupling agents leads to robust and broad peaks around 1080 cm^−1^, which can be attributed to the CO-O-CO stretching vibration of the compatibilizers. This suggests that a chemical interaction occurs between the cellulose and the coupling agents [73].

In order to complement the qualitative interpretation of the FTIR spectra, a quantitative analysis was performed by calculating the areas of the absorption peaks corresponding to specific vibrational bands (see Table 3). In particular, the area ratios A_1448_/A_1743_ (associated with CH_3_ bending and C=O stretching in PLA, respectively) and A_867_/A_1743_ (related to C–H rocking in cellulose and C=O stretching in PLA) were determined for all formulations (F0–F16). These ratios remained relatively stable, ranging from 0.21 to 0.27 and 0.060 to 0.086, respectively. This low variability suggests a uniform distribution of the cellulosic reinforcement and indicates that the incorporation of cellulose did not significantly alter the bulk chemical structure of the PLA matrix. Nevertheless, the presence of characteristic peaks and minor variations in band shape and width continue to support the occurrence of molecular interactions between PLA and the cellulosic phases. 

#### 3.2.2. Thermal Analysis

**Figure 6:**
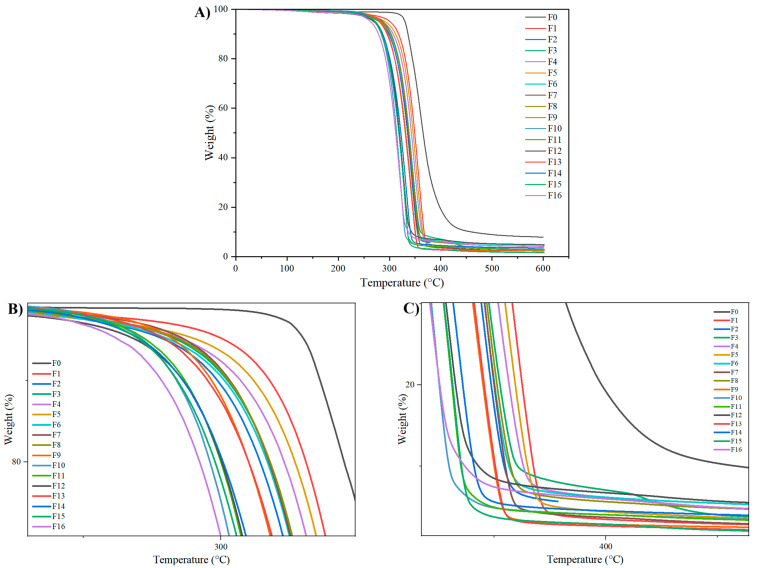
Thermogravimetric analysis of PLA and cellulose reinforced biocomposites. (**A**) complete thermogram; (**B**) initial degradation region, and; (**C**) final degradation region.

Thermal mass loss curves of the neat PLA and the biocomposites formulated are shown in Figure 6B. The degradation temperatures (T_d10_, T_d50,_ and T_d90_) and the residual char fraction at 600 °C are given in Table 4. Note that the degradation onset temperature can be influenced by the type and content of the reinforcement used, relative to pure PLA [74]. PLA was thermally stable up to 320 °C and then began to degrade rapidly in a single step. The loss of 90% of its mass was observed at approximately 460 °C, and there was a 1.4% residue at 600 °C. In contrast, thermal degradation of PLA-CPP cellulose composites initiates at temperatures in the range of 280–300 °C, approximately 10% lower than that of the PLA, leading to rapid weight losses up to 350 °C. This is primarily associated with the thermal degradation of hemicelluloses and cellulose. The degradation temperatures were lower for the biocomposites incorporating OA-KOH-treated pea pods. In a previous study using fibers obtained from sugarcane bagasse in matrices of bioepoxy and unsaturated polyester resins, chemical treatments with alkali, silane, and OA were performed to improve fiber-matrix adhesion and enhance mechanical, thermal, and physical properties. The results showed that the alkali-treated sample exhibited better thermal stability. However, no significant changes were observed with oxalic acid treatment, resulting in lower stability [75]. Without compatibilizers, the degradation temperatures decreased as the reinforcement content increased from 10 to 20 wt.%. The literature also corroborates that the treatment of cellulose with certain acids (e.g., sulfuric acid) can reduce the thermal stability of CPP celluloses and increase carbon formation at the end of the degradation process [76]. Furthermore, the decrease in the thermal stability of the composites can be attributed to the lower decomposition temperature of cellulose relative to PLA [77]. For example, Tan et al. (2021) reported that the maximum degradation temperature decreased due to the lower initial degradation temperature of microcellulose (MCC), which leads to reduced thermal stability of the composite [78]. Other contributing factors include the agglomeration and poor dispersion of cellulose in the matrix, which accelerate the diffusion of volatile decomposition products and may facilitate the propagation of oxygen in the matrix [79].

#### 3.2.3. DSC Analysis

**Figure 7:**
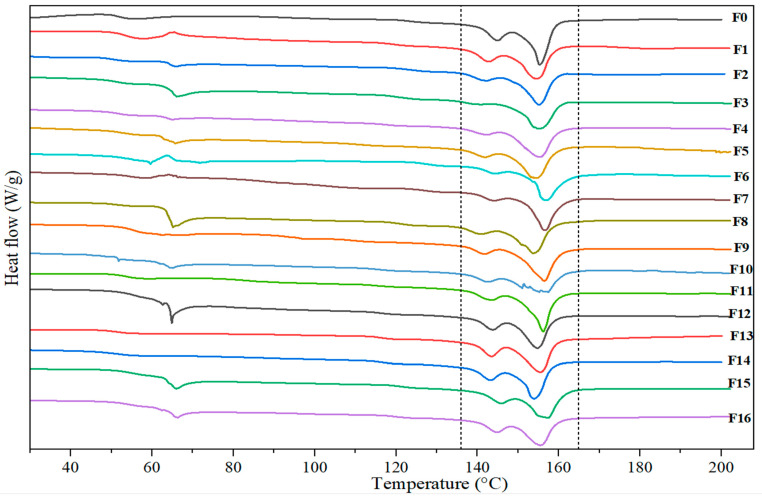
DSC thermograms of the PLA-based composites with different cellulosic content.

The DSC thermograms recorded during the first and second heating of the pure PLA and the biocomposites formulated are shown in Figure 7. The data derived from DSC analysis are given in Table 5. Thermograms exhibited a double endothermic melting peak for PLA and the PLA-CPP composites. The appearance of multiple melting peaks is common in many semi-crystalline polymers, and the literature suggests several explanations for this phenomenon, such as the presence of different crystallographic forms, crystalline layers with various thicknesses, and melting and recrystallization co-occurring [80].

During the first heating cycle, the T_g_ of both the composites and the pure PLA was very similar, ranging between 50 and 55 °C. After T_g_, a small endothermic peak was observed for both pure PLA and its biocomposites, which is a typical effect of physical aging [81,82]. No exothermic cold crystallization peak was detected for the samples annealed at 120 °C for 4 h. Regarding the degree of crystallization, it was higher than 30% for most of the biocomposites. It was also observed that, in general, the crystallinity increased as the reinforcement content went from 10 to 20 wt.%. This can be attributed to the nucleation capacity of the cellulosic reinforcements, which promotes the crystallization of PLA [83]. Additionally, the increase in χ_c_ was higher for those biocomposites incorporating pea pods treated with OA and KOH. Annealing enhances crystallinity due to PLA’s ability to crystallize between its glass transition and melting temperatures, leading to improved mechanical properties. Simmons et al. (2019) [84] developed a strategy to enhance the thermal and mechanical properties of PLA through a reactive extrusion process and the addition of a biofiller as a nucleating agent. The results indicate that annealing at 100 °C increased the crystallinity by 45% and 50% for the developed formulations [84].

#### 3.2.4. TMA Analysis

**Figure 8:**
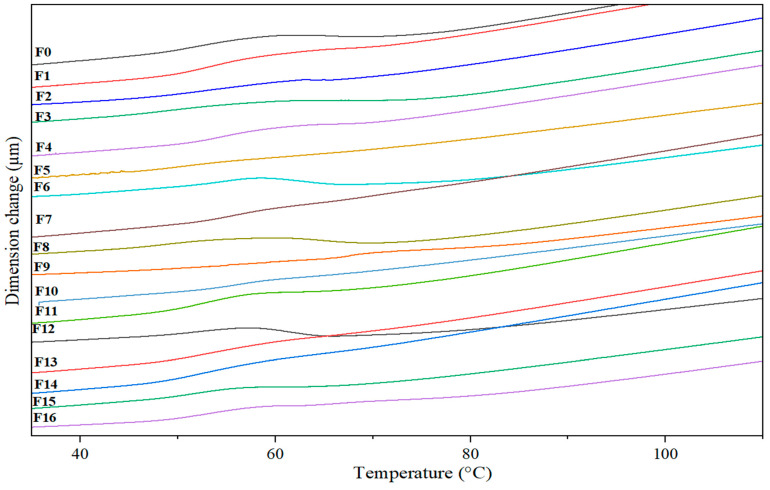
TMA thermograms of the PLA-based composites at different filler contents.

Figure 8 illustrates the effect of the cellulosic reinforcements on the thermal expansion of the biocomposites. The glass transition ranges from 50 to 56 °C, in which a maximum slope change is identified. The point where this shift change occurs corresponds to the glass transition temperature T_g_, which is similar to the one obtained by DSC for the biocomposites studied (see Table 5 and Figure 7). Table 6 lists the coefficients of thermal expansion (CTE) for each PLA and PLA-CPP composite. The pure PLA matrix exhibited a larger dimensional change as the temperature ranged from 25 to 120 °C. This is because the molecular chains within the polymer move vigorously with higher thermal energy, causing the material to expand [85]. In general, an improvement in the dimensional stability of the composites is observed, and their thermal expansion was lower below the T_g_. The CTE decreased as the content of cellulosic reinforcement increased. For instance, the CTE decreased from 95.38 for PLA to approximately 50 × 10^−6^ 1/°C^−1^ for the composite F16, reinforced with 20 wt.% cellulose. This behavior is attributed to the interfacial adhesion between the cellulosic reinforcements and the matrix in the biocomposite [86]. In this regard, this behavior is similar to the effect of adding cellulose nanofibers to a PLA matrix, which decreased the CTE by 24% compared to pure PLA [87]. In another previous study, biodegradable biocomposites based on PLA with basalt fibers (BF) or wood fibers (WF) at 7.5 or 15 wt.% were prepared by injection molding. The incorporation of both types of fibers decreased the CTE, which was attributed to the low expansion coefficients of cellulose and basalt. The lowest CTE values were recorded for PLA/15BF, with a 53% decrease compared to pure PLA [88]. PLA-based composites are mainly used in temperature ranges below T_g_. The lower CTE in these materials is advantageous for dimensional stability, helping to reduce warping and other shape changes during use, especially under thermomechanical stresses [74].

#### 3.2.5. Mechanical Behavior

**Figure 9:**
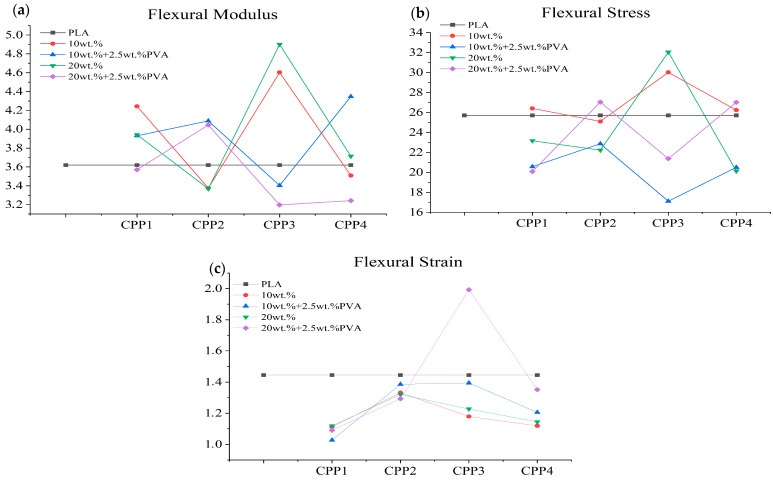
Flexural modulus (**a**), strength (**b**), and strain (**c**) of PLA biocomposites incorporating cellulosic materials obtained through acid and acid-alkaline treatments of pea pod residues.

The results of the flexural tests conducted on the PLA-CPP composites are detailed in Table A1 and illustrated in Figure 9. The flexural strength and modulus show similar trends and increase or decrease depending on the type of treatment used for the incorporated pea pod cellulose. When no PVA compatibilizer was used, the biocomposites reinforced with pea pods treated with 0.5 M OA (CPP1, CPP3) showed higher stiffness and strength compared to those treated with 0.75 M OA (CPP2, CPP4). Conversely, when PVA was included in the formulation, the biocomposites with CPP2 and CPP4 cellulosic reinforcements showed higher modulus and strength. PVA is a water-soluble, nontoxic, biocompatible, and ductile polymer. Its abundant –OH groups allow the formation of hydrogen bonds with the hydrophilic surfaces of cellulose, which can reduce agglomeration and improve interfacial adhesion [89]. These results are similar to those observed on eco-friendly PLA-MCC biocomposites prepared using two coupling agents: maleic anhydride (MAH) and maleic acid (MA). The incorporation of MAH−MA in the PLA/3 wt.% of MCC composite improved both the tensile strength and the tensile modulus; however, a decrease in elongation at break was observed. Water absorption tests demonstrated that the incorporation of MAH−MA into the PLA/MCC composite led to advantageous water barrier characteristics [72].

In general, there were no significant differences in stiffness and strength for the materials with 10 and 20 wt.% cellulosic reinforcements, except for the CPP4. In this case, using only a 10 wt.% reinforcement and PVA resulted in a higher flexural modulus but a lower strength. This can be explained by the improved crystallinity, which leads to the orientation of PLA molecular chains, crystal fragmentation, and slippage during stretching [90].

In addition to the effects of the treatments and the use of compatibilizers, note that the formulations F9 and F10, including pea pods treated with 0.5 M OA and 5 wt.% KOH, without compatibilizer, exhibited the highest flexural modulus and strength among all the formulations studied. Specifically, the average flexural modulus/strength for F9 and F10 increased by 27/17% and 35/24% compared to the PLA matrix, respectively. Suryanegara et al. (2010) studied cellulose-reinforced PLA, obtaining improvements in the modulus from 3.3 GPa to 4.0 GPa and the strength from 50.2 MPa to 60.9 MPa, while the strain at break was reduced from around 7 to 3% [91]. It is also worth mentioning that incorporating 10 wt.% pea pods treated only with 0.5 M OA (F1) increased the average flexural modulus and strength by 17% and 3%, respectively, compared to the PLA matrix. These results show that friendlier treatments could also result in significant improvements in the mechanical behavior of biocomposites such as the ones studied in this work.

On the other hand, the maximum flexural strain decreased between 10% and 20% for every one of the biocomposites prepared, except for F12. In this case, the strain at failure increased by 37% when a CPP3 cellulosic reinforcement and PVA were used.

#### 3.2.6. Water Absorption

**Figure 10:**
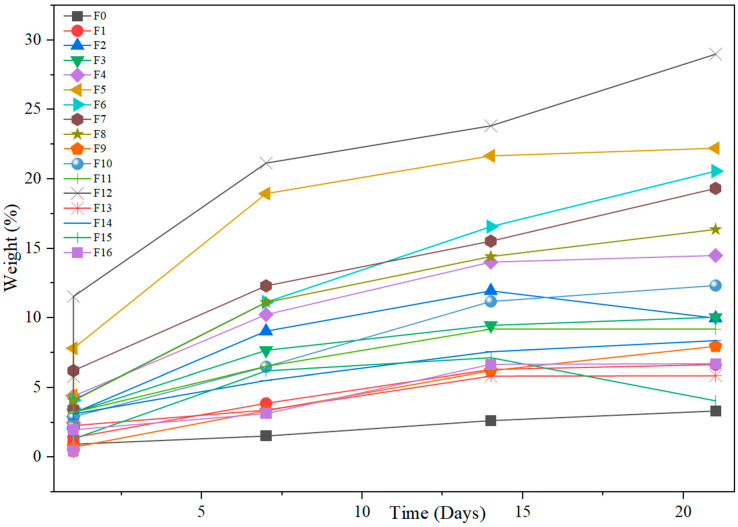
Effect of cellulosic reinforcement content and time on water absorption.

Figure 10 and Table A2 show the percentage of water absorption for PLA and PLA-CPP composites determined after 1 to 21 days of immersion. As expected, the biocomposites exhibited higher water absorption (ranging from 5.8 to 29 after 21 days) compared to neat PLA (3.3% at 21 days). In general, as the cellulosic reinforcement fraction increased, the water absorption also increased. This can be explained by considering the hydrophilic nature of the cellulosic materials, which facilitates the interaction of the biocomposites with water molecules, promoting the formation of hydrogen bonds and the subsequent absorption of water. This was also observed in other previous works. The results obtained in this work align with trends reported in previous studies on cellulose-reinforced PLA biocomposites, where water absorption increases with higher filler content due to the hydrophilic nature of cellulose. For instance, in PLA-based biocomposites reinforced with cellulose derived from durian peel (25 and 35 wt.%), the amount of water absorbed by the composites for 50 days increased with increasing cellulose content. The increase in the cellulose fraction also increased the number of hydroxyl groups (OH) observed [39]. Mahmoud et al. (2022) investigated PLA composites reinforced with microcrystalline cellulose extracted from apricot and walnut shells and showed that composites reached around 5.5% water absorption after ~23 days for samples with 10 wt.% cellulose, while pure PLA absorbed only ~1.5% under the same conditions [92]. These values are still lower than those observed in this study, where water absorption ranged from 5.8% to 29% after 21 days; the higher absorption observed here may be attributed to the combined effect of the specific morphology of pea pod-derived cellulose and the incorporation of PVA. Including PVA in the formulations increases the amount of water absorbed in the samples tested, except for the materials reinforced with pea pods treated with 0.75 M OA. This can be explained by considering that PVA possesses abundant hydroxyl groups along its molecular chains. The incorporation of cellulose fibers into the matrix allows the hydroxyl groups in both cellulose and PVA molecules to form intra- or intermolecular hydrogen bonds [93]. Cellulosic fibers absorb moisture as these hydrogen bonds are broken, enabling the -OH groups to form new hydrogen bonds with water molecules. As a result, the hydrophilic -OH groups contribute to the high-water absorption capacity of cellulosic fibers [94]. Furthermore, differences in immersion temperature (often unspecified) and reinforcement crystallinity may account for variations in the reported values. Overall, the trend of increasing water absorption with cellulose content is consistently observed across studies.

Note also that, for the biocomposites incorporating pea pods treated with 0.75 M OA, the formulations F5 and F7 with 10 wt.% reinforcement have a higher absorption compared to those in which 20 wt.% cellulose was used. This may be due to water penetrating the amorphous phase and catalyzing the hydrolytic degradation of the PLA chains. However, it also depends on the degree of crystallinity, as a high degree of crystallinity limits the hydration of the ordered chains [95].

**Figure 11:**
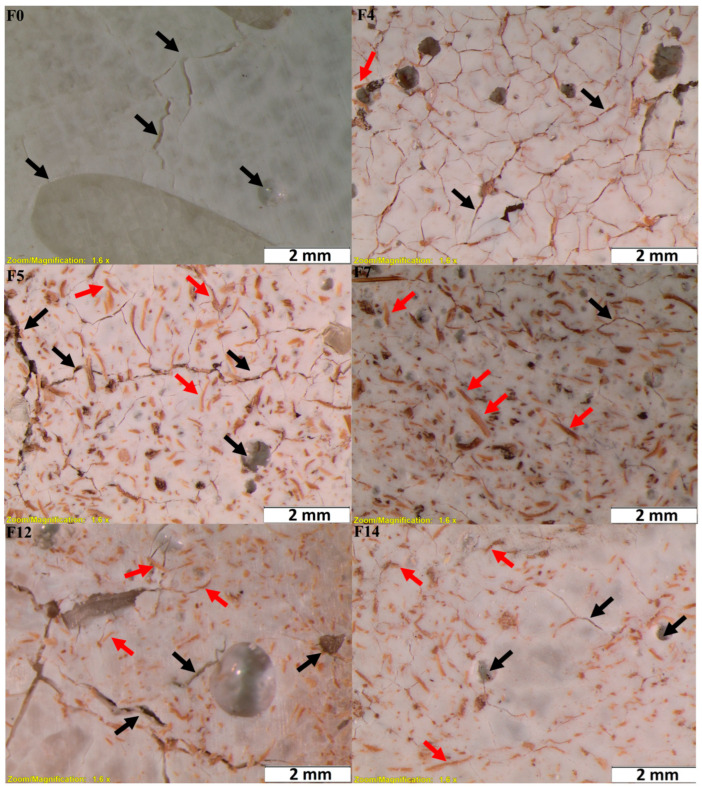
Macrographs of PLA and PLA composite formulations F0, F4, F5, F7, F12, and F14 after 21 days. Black arrows: fractures or voids. Red arrows: cellulose fibers.

PLA degradation occurs at a faster pace when the temperature is higher than its glass transition temperature (T_g_~50–60 °C). However, it is worth mentioning that water absorption also determines the hydrolytic degradation of PLA and its composites [96]. In this work, all the biocomposite samples showed degradation from the seventh day on, except for pure PLA. PLA degrades by simple hydrolysis, which depends on moisture content, molecular weight, crystallinity, molar mass, temperature, and water absorption [94]. As shown in Figure 11, after 21 days, the biocomposite samples exhibited a very porous, fragile structure and typically lost integrity during handling. For that reason, the test could not continue beyond this point.

A visual inspection suggests that the formulation has a significant influence on the presence and distribution of cracks and voids. For instance, the control sample F0 (pure PLA) exhibits extensive cracking and void formation (black arrows), which is consistent with the lack of reinforcement. Sample F5, containing 10% cellulose treated with 0.75 M oxalic acid but without a compatibilizer, shows numerous voids and irregular fiber dispersion, indicating poor interfacial adhesion between fiber and matrix. In contrast, sample F7 (10% cellulose treated with 0.75 M oxalic acid and 2.5% PVA compatibilizer) displays the most favorable morphology: the fibers (red arrows) appear well dispersed and embedded in the PLA matrix, with minimal cracking or voids. This suggests a synergistic effect between the fiber surface treatment and the compatibilizer, resulting in improved fiber–matrix interaction. Other formulations, such as F12 and F14, which include more intense treatments (oxalic acid + KOH) and/or higher fiber content (20%), also benefit from improved morphology compared to F0 and F5 but still exhibit slightly more defects than F7. This could be due to either the increased fiber loading or the less optimal interfacial compatibility.

While the increase in water absorption observed in the biocomposites may limit their use in moisture-sensitive applications (e.g., structural components or long-term food packaging), it could be acceptable or even beneficial in short-lifetime applications, such as agricultural mulch films, compostable trays, or disposable packaging, where biodegradability is desired. Moreover, although no surface modification of the cellulose was performed in this study, several works report that chemical treatments such as acetylation, silanization, or grafting with hydrophobic groups can significantly reduce water absorption by lowering the availability of surface hydroxyl groups on cellulose fibers [94,97,98]. These surface modification strategies are promising and will be considered in future work to improve the moisture resistance of these biocomposites and broaden their application range.

## 4. Conclusions

The use of agroindustrial residues to obtain valuable cellulosic materials constitutes an alternative to mitigate the environmental problems derived from their improper disposal. If these cellulosic materials can be conveniently incorporated as reinforcements for high-performance biocomposites, it is also possible to contribute to reducing the negative impacts of conventional plastics. In this work, we demonstrated that it is feasible to devise high-yield chemical treatments to obtain cellulose from abundant, widely available, inexpensive lignocellulosic residues such as pea pod husks. These treatments offer an opportunity for the valorization of the residues and can be based on the use of low-concentration organic acids, such as 0.5 or 0.75 M OA. This treatment allows us to obtain moderately hydrophilic cellulose with relatively high degradation temperatures (352 or 341 °C). This cellulose can be well dispersed in biodegradable bio-based PLA matrices by extrusion and easily formed by compression molding. The resulting biocomposite specimens are moderately crystalline (29–36%), have glass transition temperatures similar to that of PLA (51–55 °C), and exhibit high degradation temperatures (353–339 °C). They also show higher flexural stiffness (up to 17%), similar strength, and flexural strain at break compared to the pure PLA matrix.

The amount of water absorbed at 50 °C changes significantly (up to 234% of the initial dry weight) depending on the concentration of the oxalic acid, providing opportunities to tailor the composite degradation. On the other hand, if the OA is combined with an alkaline 5% *w*/*v* KOH treatment, the cellulose yields are around 64%, the crystallinity reaches values around 92%, while the degradation temperature decreases slightly (T_max_ 328 °C). The biocomposites incorporating these celluloses show T_g_, χ_c_, and water absorption similar to those obtained with the pea pods treated only with OA. The advantage of these materials is in the mechanical performance, since the flexural modulus and strength can increase up to 35% and 25%, reaching values up to 4.9 GPa and 32.1 MPa, respectively. These values are similar to those obtained using cellulosic reinforcements derived from treatments that include more potentially harmful chemicals. Therefore, the results obtained are encouraging and provide new perspectives for the use of friendlier treatments to obtain moderately crystalline cellulose that can impart good mechanical performance and tailorable degradation to biocomposites.

The incorporation of cellulose from pea pod waste into a PLA matrix improves mechanical strength and water absorption, demonstrating its potential as a sustainable alternative to conventional plastics. These composites are particularly well-suited for biodegradable packaging, agricultural films, and short-life disposable items, where renewable origin, reinforcement, hydrophilicity, and biodegradability are essential. The valorization of agro-industrial waste further highlights the environmental and practical significance of these materials.

## Figures and Tables

**Table 1:** Formulations of the PLA biocomposites studied.

Formulation	Composition (wt.%)			
	PLA	Cellulose Reinforcement	Treatment	PVA Compatibilizer
**F0**	100	0	-	0
**F1**	90	10	CPP1 0.5 M of OA	0
**F2**	80	20		0
**F3**	87.5	10		2.5
**F4**	77.5	20		2.5
**F5**	90	10	CPP2 0.75 M of OA	0
**F6**	80	20		0
**F7**	87.5	10		2.5
**F8**	77.5	20		2.5
**F9**	90	10	CPP3 0.5 M of OA + 5% of KOH	0
**F10**	80	20		0
**F11**	87.5	10		2.5
**F12**	77.5	20		2.5
**F13**	90	10	CPP4 0.75 M of OA + 5% of KOH	0
**F14**	80	20		0
**F15**	87.5	10		2.5
**F16**	77.5	20		2.5

**Table 2:** Degradation onset and maximum temperatures, as well as char residues of raw and treated pea pods, determined from TGA analyses.

Samples	Crystallinity Index (%)	$T_{onset}$ °C	$T_{max}$ °C	Char Residue at 800 °C (%)
**Raw material**	-	227.01	315.77	26.29
**CPP1**	65.59	283.91	351.60	15.60
**CPP2**	64.41	283.11	341.30	14.21
**CPP3**	91.14	285.63	328.37	19.87
**CPP4**	92.62	284.32	327.93	18.73

**Table 3:** Quantitative analysis of FTIR absorption peak area ratios for PLA/cellulose composites.

Formulation	A1448/A1743	A867/A1743
**F0**	0.250	0.0810
**F1**	0.266	0.0857
**F2**	0.217	0.0684
**F3**	0.257	0.0846
**F4**	0.220	0.0654
**F5**	0.223	0.0670
**F6**	0.245	0.0786
**F7**	0.222	0.0677
**F8**	0.238	0.0667
**F9**	0.252	0.0820
**F10**	0.219	0.0662
**F11**	0.231	0.0682
**F12**	0.248	0.0790
**F13**	0.255	0.0831
**F14**	0.225	0.0703
**F15**	0.214	0.0606
**F16**	0.243	0.0731

**Table 4:** Thermal degradation of biocomposites with different cellulosic reinforcements.

Sample	T_d10_ (°C)	T _d50_ (°C)	T _d90_ (°C)	Char Residue at 600 °C (%)
**F0**	331.09	365.5	459.6	1.416
**F1**	317.7	349.17	367.46	0.702
**F2**	298.77	335.36	354.39	0.654
**F3**	302.43	338.25	360.42	0.5629
**F4**	307.2	342.73	363.02	1.152
**F5**	311.93	346.06	364.89	0.416
**F6**	301.25	336.89	356.51	1.310
**F7**	303.2	337.02	354.9	0.402
**F8**	302.79	337.85	357.26	0.806
**F9**	296.64	330.7	349.92	0.593
**F10**	283.96	313.85	329.73	0.543
**F11**	286.89	318.79	335	0.801
**F12**	286.24	319.54	342.95	1.478
**F13**	290.11	328.12	346.7	1.83
**F14**	288.10	325.49	344.48	2.11
**F15**	284.22	318.22	335.23	0.438
**F16**	278.48	312.03	336.83	1.156

**Table 5:** Thermal properties of PLA and PLA-CPP composites obtained by DSC.

Samples	1st Heating				
	T_g_ (°C)	T_m_ (°C)		∆H_m_ (J/g)	χ_c_ (%)
		T_m1_	T_m2_		
**F0**	51.87	140.63	152.96	31.20	33.55
**F1**	53.46	138.04	149.68	29.28	34.98
**F2**	50.26	136.46	149.99	22.35	30.04
**F3**	51.74	134.57	150.52	21.52	27.47
**F4**	51.94	140.95	148.21	23.97	29.86
**F5**	53.22	137.08	149.04	25.29	28.64
**F6**	55.14	139.98	154.02	20.61	33.99
**F7**	53.99	139.60	152.81	21.93	25.33
**F8**	51.19	135.83	149.11	25.51	30.43
**F9**	55.41	137.19	149.66	26.35	30.48
**F10**	51.68	137.59	150.41	23.99	35.42
**F11**	53.15	137.89	152.45	25.97	29.48
**F12**	56.57	139.43	149.60	24.59	36.03
**F13**	51.75	139.29	149.13	28.51	29.38
**F14**	51.88	138.57	151.14	25.10	38.32
**F15**	55.05	141.16	152.01	26.42	32.09
**F16**	53.87	140.33	150.11	23.89	36.66

**Table 6:** Glass transition temperature and CTE of the PLA and the biocomposites.

Sample	T1 (°C)	α_1_ [μm (m·°C)^−1^] 30–50 °C
**F0**	49.96	95.38
**F1**	49.22	90.38
**F2**	49.33	75.53
**F3**	46.02	85.83
**F4**	47.94	78.75
**F5**	44.53	77.71
**F6**	50	76.01
**F7**	49.65	83.35
**F8**	46.55	65.04
**F9**	49.82	76.71
**F10**	50	81.54
**F11**	49.31	90.97
**F12**	48.92	57.72
**F13**	50	92.04
**F14**	50	95.33
**F15**	48.37	81.75
**F16**	48.15	50.48

**Figure A1:**
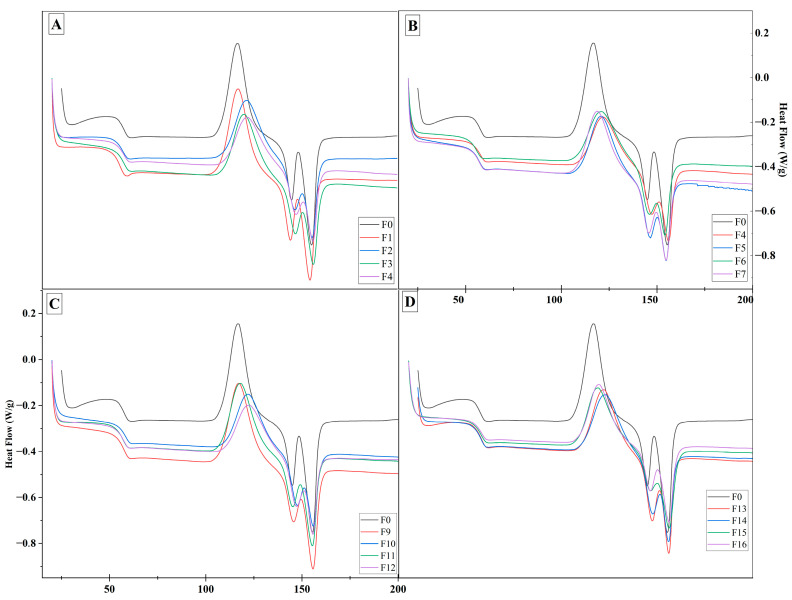
DSC thermograms of the second heating for PLA and PLA-based composites with different cellulosic content. (**A**) CPP1 treatment; (**B**) CPP2 treatment; (**C**) CPP3 treatment; (**D**) CPP4 treatment.

**Figure A2:**
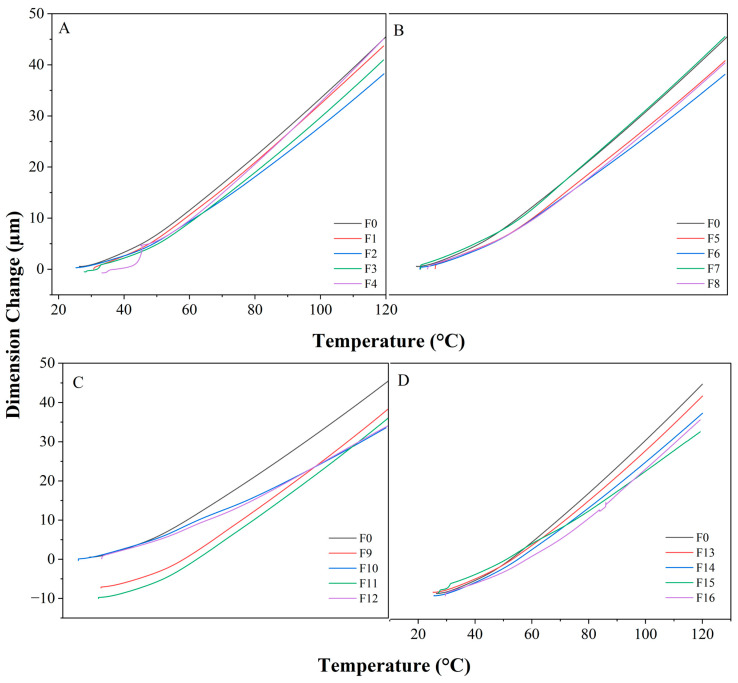
TMA thermograms of the second heating for PLA-based composites at different filler content. (**A**) CPP1 treatment; (**B**) CPP2 treatment; (**C**) CPP3 treatment; (**D**) CPP4 treatment.

## Data Availability

The original contributions presented in this study are included in the article. Further inquiries can be directed to the corresponding author.

## Appendix A

**Table A1:** Mechanical properties of neat PLA and its biocomposites with cellulosic reinforcements.

Samples	Flexural Modulus (GPa)	Flexural Strength (MPa)	Flexural Strain (%)
**F0**	3.620 ± 0.102	25.710 ± 3.973	1.445 ± 0.093
**F1**	4.244 ± 0.249	26.407 ± 3.179	1.112 ± 0.057
**F2**	3.942 ± 0.074	23.168 ± 2.536	1.119 ± 0.046
**F3**	3.931 ± 0.404	20.576 ± 2.297	1.027 ± 0.117
**F4**	3.571 ± 0.176	20.086 ± 1.832	1.091 ± 0.119
**F5**	3.375 ± 0.417	25.104 ± 3.583	1.332 ± 0.131
**F6**	3.369 ± 0.189	22.225 ± 1.609	1.320 ± 0.059
**F7**	4.088 ± 0.201	22.863 ± 2.820	1.386 ± 0.224
**F8**	4.045 ± 0.322	27.036 ± 0.867	1.293 ± 0.096
**F9**	4.603 ± 0.750	30.018 ± 1.085	1.179 ± 0.150
**F10**	4.900 ± 0.215	32.051 ± 2.997	1.228 ± 0.085
**F11**	3.403 ± 0.154	17.108 ± 1.574	1.395 ± 0.316
**F12**	3.197 ± 0.568	21.371 ± 0.387	1.993 ± 0.499
**F13**	3.509 ± 0.143	26.235 ± 0.733	1.119 ± 0.040
**F14**	3.713 ± 0.216	20.137 ± 1.086	1.144 ± 0.253
**F15**	3.619 ± 0.127	19.890 ± 2.474	1.131 ± 0.145
**F16**	3.700 ± 0.234	18.969 ± 2.084	1.117 ± 0.060

**Table A2:** Water absorption of neat PLA and composites reinforced with cellulose.

Water Absorption (%)					
Time (Days)	1	2	7	14	21
**F0**	0.708	0.934	1.522	2.631	3.319
**F1**	1.062	1.411	3.875	6.291	6.637
**F2**	2.271	3.166	9.040	11.944	9.975
**F3**	1.908	3.197	7.665	9.457	10.050
**F4**	2.472	4.395	10.235	14.016	14.494
**F5**	4.453	7.806	18.951	21.669	22.222
**F6**	3.505	4.091	11.132	16.568	20.570
**F7**	3.486	6.203	12.306	15.526	19.326
**F8**	2.320	4.061	11.114	14.417	16.368
**F9**	0.442	0.724	3.340	6.207	7.953
**F10**	2.237	2.907	6.483	11.178	12.331
**F11**	2.211	3.225	6.515	9.196	9.196
**F12**	5.795	11.534	21.149	23.833	28.992
**F13**	1.955	2.273	3.388	5.822	5.844
**F14**	2.717	3.103	5.509	7.560	8.359
**F15**	0.285	1.318	6.185	7.130	4.048
**F16**	0.459	1.973	3.128	6.656	6.698

**Table A3:** Mechanical performance of PLA and composites reinforced with cellulose.

Sample	Relation	Flexural Modulus (GPa)	Flexural Strength (MPa)	Flexural Strain (%)	Results	References
**PLA**	100	3	8.4	--	The flexural modulus and strength increase by 25% and 80%, respectively, with 20% of MFC. Flexural strength increases by 130% with 1% CNC. At 5% CNC, the flexural modulus increases to 175%	[10]
**PLA-cellulose microfibers (MFC)**	80:20	3.8	16.5	--		
**PLA-MFC-cellulose nanocrystals (CNC)**	80:19:1 80:15:5	5.3 5.7	19.2 26.5	--		
**PLA**	100	1.17	15.24	40.30	At 1% CNF-Ac into PLA films, the strain increased by more than 60 percent. CNF-Ac to 3%, the tensile strength and tensile modulus increase.	[99]
**PLA/acetylated cellulose nanofiber (CNF-Ac)-1**	1% CNF-Ac	1.13	16.70	64.77		
**PLA/CNF-Ac-3**	3% CNF-Ac	1.20	33.06	188.86		
**PLA/microcrystalline cellulose (MCC)-3**	3% MCC	1.19	15.88	38.47		
**PLA**	100		14.72	10.32	At 1 wt% NFC, the tensile strain increases to 87.9% compared with PLA. When NFC is at 5 wt%, tensile strength reaches a 98.8% boost.	[90]
**PLA/nanofibrillated cellulose (NFC)-3wt%**	3 wt% NFC		27.08	17.95		
**PLA/NFC-5wt%**	5 wt% NFC		29.26	18.67		
**PLA/NFC-10wt%**	10 wt% NFC		26.30	15.71		
**PLA**	100	3.3	57.7	6.8	20 wt% of MFC improved the modulus of amorphous PLA from 3.3 GPa to 5.2 GPa and the tensile strength.	[100]
**PLA/microfibrillated cellulose (MFC) 3wt%**	3 wt% MFC	3.8	61.4	2.7		
**PLA/MFC 5wt%**	5 wt% MFC	3.9	63.4	2.5		
**PLA/MFC 10wt%**	10 wt% MFC	4.5	65.4	2.2		
**PLA/MFC 20wt%**	20 wt% MFC	5.2	70.2	1.9		
